# Retinal Vessel Diameter Changes in Relation to Dark Adaptation and Acute Hyperglycemia

**DOI:** 10.1155/2018/7064359

**Published:** 2018-09-18

**Authors:** Per Kappelgaard, Stig K. Holfort, Oliver N. Klefter, Michael Larsen

**Affiliations:** ^1^Department of Ophthalmology, Rigshospitalet, Copenhagen, Denmark; ^2^Faculty of Health and Medical Sciences, University of Copenhagen, Copenhagen, Denmark

## Abstract

The purpose of this experimental clinical study was to assess the effects of dark adaptation and acute changes in glycemia on retinal vessel diameters in men. The study included 14 patients (mean age 63 years, range 48–74 years) with type 2 diabetes mellitus and minimal or no diabetic retinopathy. Retinal vessel diameters were assessed using infrared photography before and after dark adaptation, first while fasting and then at peak hyperglycemia during an oral glucose tolerance test (OGTT). Dark adaptation was accompanied by retinal vasodilatation, both during fasting (mean glycemia 7.6 ± 1.7 mM) and postprandial hyperglycemia (15.7 ± 4.2 mM). When fasting, the increase in vein diameter during dark adaptation was 2.0% after 20 min (*P*=0.018) and 2.9% after 40 min (*P*=0.010). When subjects were hyperglycemic, the increase during dark adaptation was 2.8% for retinal vein diameters (*P*=0.027) and 2.0% for retinal artery diameters after 20 min (*P*=0.002) and 1.7% for retinal artery diameters after 40 min (*P*=0.022). For identical conditions of light/dark adaptation, retinal vessels were dilated when subjects were fasting compared to postprandial hyperglycemia. Thus, darkness and fasting were both associated with retinal vasodilation in this short-term experiment in patients with type 2 diabetes. Future studies should determine whether both the stimuli of vasodilation lead to retinal hyperperfusion, which would support that they may be involved in the aggravation of diabetic retinopathy.

## 1. Introduction

Development of diabetic retinopathy and progression to proliferative diabetic retinopathy are accompanied by dilation of the retinal veins [1–3]. It is of interest to identify risk factors for retinal vasodilation, especially the ones that are modifiable, because the information thus obtained can be used in the search of targets for intervention. Factors of specific interest are the adaptation of the eye to changes in ambient light and its adaptation and changes in glucose levels, notably in diabetes.

Dark adaptation is accompanied by decreasing oxygen tension and increasing lactate production in the retina [4–7] and an increase in retinal blood flow, part of which might be mediated by blood vessel dilation [8–10].

Retinal vessel dilation occurs in response to experimental hypoxia and flicker stimulation [11–13], and lower glycemia suppresses retinal electroretinographic signaling and dark adaptation. Hence, there is a reason to suspect that hypoglycemia may dilate retinal vessels [14–17]. While some experiments have found abnormal autoregulation of blood flow in the retina in diabetes, the relation between light exposure, glycemia, and blood flow outlined above may also exist in diabetes, and there is a reason to suspect that it may contribute to the development and progression of retinopathy in diabetes [18–21].

The objective of the present study was to examine the acute concomitant effects of dark adaptation and changes in glycemia levels on retinal vessel diameters in patients with type 2 diabetes.

## 2. Materials and Methods

This experimental clinical study included 14 patients with type 2 diabetes mellitus, 9 men and 5 women, aged 48–74 years (mean ± SD 62.6 ± 7.5 years; Table 1). Best-corrected visual acuity (Snellen) was 0.9 or better in all eyes. The patients had mild (ETDRS level < 35) or no diabetic retinopathy and no study eye had macular edema. Exclusion criteria were eye diseases other than diabetic retinopathy, untreated arterial hypertension, and major systemic diseases other than diabetes. All 14 patients were recruited from the internal medicine outpatient clinic of Rigshospitalet; 13 were treated with one or more oral antidiabetic agents, while one was treated by diet alone. Four patients were treated by insulin. The study was approved by the Danish National Committee on Biomedical Research Ethics and conducted with the participants' informed consent according to the Helsinki Declaration II. The study was registered at Clinicaltrials.gov (NCT01136902).

Infrared scanning laser fundus photography (Spectralis HRA + OCT, Heidelberg Engineering, Heidelberg, Germany) was made over a 55-degree angle centered on the optic disc with a nominal resolution of 768 × 768 pixels. A computerized dark adaptometer (AdapDx, MacuLogix, Inc., Middletown, PA, USA) was used in three patients to verify that full dark adaptation had been obtained at the end of the examination [22].

Oral glucose tolerance testing (OGTT) was performed by having the patients ingest 75 g of glucose dissolved in 250 mL of water. The subsequent hyperglycemia was expected to reach its peak after 1.5 hours. The experiments were made between 8 am to 12 pm after an overnight fast and the patients not having taken their antidiabetic agents since the evening before.

Study procedures included determination of best-corrected visual acuity (Snellen), transfoveal optical coherence tomography (Cirrus™ HD-OCT, Carl Zeiss Meditec AG, Jena, Germany), infrared fundus photography (Spectralis® HRA + OCT, Heidelberg Engineering, Heidelberg, Germany), and venous blood analysis for HbA_1c_, sodium, and potassium. Pupil dilation was made with phenylephrine hydrochloride 10% and tropicamide 1%. The study eye of each patient was selected randomly except in one patient where one eye was excluded because of diabetic macular edema.

**Figure 1:**
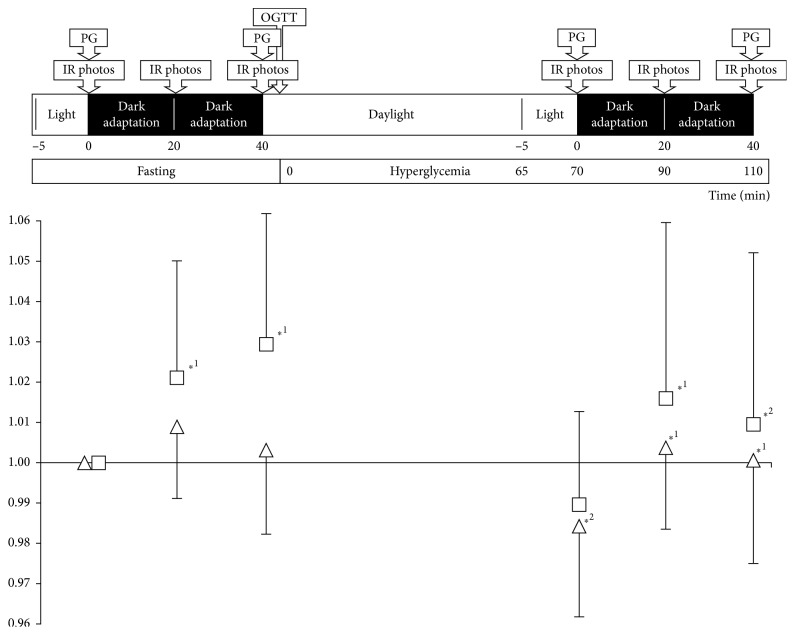
Retinal vessel diameter data indexed to baseline (triangles: arteries and squares: veins) in 14 patients with type 2 diabetes who were fasting (glycemia 7.6 ± 1.7 mM) and adapted to room light at baseline (0 minutes). This was followed by a cycle of dark adaptation and fundus photography, during which infrared fundus photography, which had also been made at baseline, was repeated twice. Daylight was reintroduced after 40 minutes and then, 5 min later, participants ingested 75 g of glucose dissolved in water. After 70 more minutes when glycemia had increased to 15.7 ± 4.2 mM, infrared photographs were recorded in daylight, after which a second round of dark adaptation and photography was reinitiated. PG, plasma glucose; OGTT, oral glucose tolerance test; IR, infrared. Values greater than 1 indicate vessel dilation and values lower than 1 indicate vessel contraction. One-sided error bars indicate 1 standard deviation. Asterisk *∗*^1^ indicates *P* < 0.05 compared to the nearest predark adaptation baseline, fasting, or hyperglycemic. Asterisk *∗*^2^ indicates *P* < 0.05 for a given time point during the second (hyperglycemic) dark adaptation cycle compared to the first (fasting) dark adaptation cycle.

Study sessions followed a fixed time schedule (Figure 1), comprising two dark adaptation cycles beginning each with infrared fundus photography in normal room light (approximately 200 lux) after 5 minutes of adaptation and continuing with a second set of images after 20 min of dark adaptation and finally a third set of images after 40 min of dark adaptation. The first cycle was made with subjects fasting, whereas the second cycle was started 65 min after the ingestion of the OGTT meal and thus made during postprandial hyperglycemia. Capillary blood glucose concentration was measured (Precision Xceed reagent-strip and reading device, Medisense Products, Abbott Laboratories, Witney, Oxon, United Kingdom) immediately before and after the two dark adaptation cycles. The means of the two pairs of glycemia values were used for data analysis.

Vessel diameters were assessed using a semiautomated algorithm as previously described [23–25]. Central retinal artery equivalent (CRAE) and central retinal vein equivalent (CRVE) diameters were estimated from the individual peripapillary vessel diameters according to the method of Knudtson et al. [26], in this case measured on the infrared fundus photographs.

The same six arteriolar and six venular trunk vessel segments were analyzed at a distance of one half to one disc diameter from the optic disc. Vessel segments were measured along their entire length within this interval if no crossing or branching was present. Trunk segments were preferred to branches unless the trunk was shorter than 80 *µ*m. The diameters of the central vessel equivalents were then calculated in the following manner:

(1) $$\begin{array}{c} \text{arterioles}:\;\;\hat{W}=0.88\times {\left(w_{1}^{2}+w_{2}^{2}\right)}^{1/2}, \end{array}$$

(2) $$\begin{array}{c} \text{venules}:\;\;\hat{W}=0.95\times {\left(w_{1}^{2}+w_{2}^{2}\right)}^{1/2}, \end{array}$$

where *w*_1_ is the width of the narrower branch, *w*_2_ is the width of the wider branch, *W* is the estimate of the width of the parent trunk arteriole or venule, and the constant is the branching coefficient. Vessel diameters, as measured or derived from equations (1) or (2), were paired and used to calculate trunk vessel width until only one number is left representing the diameter of an idealized central retinal artery or vein and named CRAE or CRVE [26].

Values for CRAE and CRVE were calculated from the three best photographs from each time point and the mean of the three values used for statistical analysis. The photographs were ranked based on sharpness, disc centration, and evenness of illumination.

The statistical analysis included two-sample paired *t*-tests of differences between fasting and hyperglycemic parameters and between light-adapted and dark-adapted parameters. Measurements are expressed as means ± standard deviations (mean ± SD) and 95% confidence intervals (CI_95_). An operational level of statistical significance of *P* ≤ 0.05 is used in the description of the results without correction for multiple testing. Additionally, mixed model analysis for repeated measurements with Tukey-adjusted comparisons (SAS 9.4, SAS Institute, Carey, NC, USA) was used to model the relative contributions of glycemia and dark adaptation to the variations in CRAE and CRVE.

## 3. Results

Mean plasma glucose concentrations were 7.6 ± 1.7 mM during the fast and rose to 15.7 ± 4.2 mM during postprandial hyperglycemia (*P* < 0.01). From the fasting baseline to hyperglycemia, in both cases before dark adaptation was begun, retinal artery diameters decreased significantly from 170.7 ± 14.8 *µ*m to 168.0 ± 13.8 *µ*m (*P*=0.025), whereas only a numerical reduction in vein diameters was seen, from 227.0 ± 21.0 *µ*m to 224.5 ± 20.8 *µ*m (*P*=0.127; Table 2; Figure 1).

While fasting, there was little change in retinal artery diameters during dark adaptation, but from the more constricted hyperglycemic baseline, dark adaptation was accompanied by a 2.0% increase in retinal artery diameter, from 168.0 ± 13.8 *µ*m to 171.4 ± 14.3 *µ*m at 20 min (*P*=0.002) and 170.8 ± 15.0 *µ*m or 1.7% more than that before dark adaptation at 40 min (*P*=0.022; Table 2).

While fasting, the retinal veins had dilated by 2.0% after 20 minutes, from 227.0 ± 21.0 *µ*m to 231.5 ± 21.0 *µ*m (*P*=0.018) and by 2.9% after 40 min, to 233.6 ± 23.1 *µ*m (*P*=0.010; Table 2; Figure 1). The retinal veins dilated, numerically, during hyperglycemic dark adaptation, this time by 2.8%, from a hyperglycemic daylight baseline of 224.5 ± 20.8 *µ*m to 230.7 ± 25.4 *µ*m after 20 minutes (*P*=0.026; Table 2) and 229.2 *µ*m or 2.1% after 40 minutes (*P*=0.072).

In a mixed model analysis covering the entire data set in a single model, we found nonsignificant interactions between glycemic state and dark adaptation, hence implying independent effects. Dark adaptation had a highly significant effect on both CRAE and CRVE (*P*=0.001 and *P* < 0.0001, respectively), leading to increases in vessel diameters in both the fasting condition and during hyperglycemia (Figure 1). Hyperglycemia was generally characterized by numerically lower CRAE and CRVE, reflected by statistical tendencies in the mixed model analysis (*P*=0.10 and *P*=0.08).

## 4. Discussion

This experimental study of retinal vessel diameter variation during acute hyperglycemia and dark adaptation in patients with type 2 diabetes found that both dark adaptation and low levels of glycemia were associated with wider retinal vessel diameters. The combined assessment of the two factors was made possible by infrared digital fundus photography, which does not disturb dark adaptation.

A tentative explanation is that dark adaptation leads to vessel dilation because oxygen tension in the retina is lower in the dark-adapted state than in daylight and that hypoglycemia leads to vessel dilation because it reduces the energy substrate supply to the retina. Presumably, but not actually documented by our experiments, vasodilation compensates for energy deficits secondary to hypoxia and hypoglycemia by increasing blood flow and substrate supply to the retina. The underlying mechanisms may obviously be complicated, if extraocular responses are elicited by changes in glycemia. Thus, plasma insulin, which should change during the glucose tolerance test, was not measured in this study, but given that the patients had type 2 diabetes, they will have varying levels of residual insulin production and insulin sensitivity. Insulin is generally considered to have vasodilator effects [27], so if plasma insulin influenced the study, it likely acted to limit the vasoconstrictive effect of hyperglycemia by the endogenous postprandial rise in insulin secretion [27, 28].

Our assumptions are supported by the demonstration by Grunwald et al. that retinal vasodilation is associated with increased retinal blood flow [29] and that dark adaptation increases retinal volumetric blood flow in healthy subjects by 62–71% [8].

Retinal vasodilation during dark adaptation is consistent with dark adaptation inducing an increase in metabolic activity of the retina [7, 30]. Increased metabolism may hypothetically promote the progression of diabetic retinopathy [31, 32] because accompanying vasodilation and hyperperfusion lead to increased shear stress on the vascular endothelium. Relative hypoglycemia, by inducing vasodilation, may have a comparable effect. This may help explain why improved metabolic control in diabetes can lead to short-term acceleration of retinopathy progression, despite its long-term benefits [33, 34]. Finally, our findings support the suggestion that nocturnal vasodilation may explain why diabetic macular edema tends to be more pronounced in the morning than later in the day [35, 36].

The vasoconstricting effect of hyperglycemia may hypothetically be driven by the ability of the retina to balance its metabolic needs by increasing glycolysis and lactate production, while decreasing oxidative phosphorylation and CO_2_ production [12]. The present study provides information only about the net result, however, and not about its constituent components.

Limitations of this study include the lack of inclusion of a group of healthy subjects. This might determine whether the characteristics in diabetes are an extension of normal physiology induced by abnormally high postprandial rises in blood glucose in diabetes or whether the physiological characteristics or the retina has been adjusted, rearranged, or disrupted by elevated and unstable glycemia or other aspects of diabetes. Additionally, a larger study population might have enabled analysis of the relation of vessel diameter changes to age, blood pressure, HbA_1c_, and other variables of interest. It should be noted that our results were obtained by summarizing observations from a group of patients. We have not shown that neither qualitative nor numerical responses can be used to define any clinically relevant characteristics of patients with diabetes.

In summary, this study of patients with type 2 diabetes found that dark adaptation was accompanied by retinal vasodilation, both during normoglycemia and hyperglycemia. Furthermore, the retinal vessels were dilated during normoglycemia compared to postprandial hyperglycemia. The study supports that the occasional flare-up of diabetic retinopathy after rapidly introduced and strictly maintained relative hypoglycemia [33, 34] may be precipitated by retinal vasodilation and that rod photoreceptor activation may promote worsening of diabetic retinopathy [32]. Our interpretations can be tested in studies that include assessment of perfusion rates and oxygenation, preferably with enough participants and a long enough period of observation to determine if individual vessel diameter responses can predict retinopathy progression.

## Figures and Tables

**Table 1:** Clinical characteristics of patients with type 2 diabetes.

Mean ± SD	
Age (years)	62.6 ± 7.5
Sex (male/female)	9/5
Duration of diabetes (years)	6.4 ± 5.0
Systolic blood pressure (mmHg)	131 ± 17
Diastolic blood pressure (mmHg)	76 ± 9
HbA_1c_ (%)	7.3 ± 1.0
Visual acuity (Snellen)	1.0 ± 0.1
PG fasting (mM)	7.6 ± 1.7
PG 1.5-hour OGTT (mM)	15.7 ± 4.2

PG, plasma glucose; OGTT, oral glucose tolerance test.

**Table 2:** Fasting and hyperglycemic retinal vessel diameters in patients with type 2 diabetes.

Patient	Fasting						Hyperglycemia					
	Light		Dark adaptation				Light		Dark adaptation			
	0 min		20 min		40 min		0 min		20 min		40 min	
	CRAE	CRVE	CRAE	CRVE	CRAE	CRVE	CRAE	CRVE	CRAE	CRVE	CRAE	CRVE
1	191.0	257.0	189.4	265.7	189.7	271.3	181.2	249.8	189.8	274.6	194.2	269.2
2	175.0	222.2	176.8	231.4	170.9	225.7	169.5	219.6	171.6	227.2	168.8	225.0
3	181.7	234.3	182.5	239.7	191.7	253.6	186.8	239.0	190.3	256.4	189.2	255.7
4	160.3	220.8	166.2	232.2	162.4	235.8	154.7	222.1	163.1	230.4	162.7	227.6
5	176.5	245.6	176.6	245.7	177.4	252.2	176.7	248.0	176.6	247.3	180.6	246.0
6	169.7	246.0	173.2	248.3	174.9	264.1	169.2	248.4	173.3	264.5	173.5	263.7
7	150.7	189.7	153.1	203.4	153.7	199.0	147.5	184.4	154.2	196.8	151.5	195.4
8	178.5	225.5	178.6	225.9	173.5	226.3	174.9	220.7	180.6	221.2	178.3	220.8
9	142.8	206.7	145.0	206.4	144.3	205.8	141.0	199.1	140.7	194.5	139.7	196.3
10	190.2	250.0	187.8	248.6	184.2	243.3	184.1	244.4	182.7	238.5	181.1	234.7
11	153.7	214.1	155.1	217.6	154.3	217.4	155.8	216.7	154.9	215.5	157.2	217.6
12	185.8	245.6	181.7	239.4	180.6	240.2	177.4	230.5	182.5	237.0	181.7	235.5
13	167.9	192.6	171.0	194.3	170.0	197.3	165.3	195.1	168.6	192.2	164.0	192.3
14	166.6	227.2	172.7	242.8	168.6	238.8	167.3	225.8	170.0	234.0	168.9	228.7
MEAN	**170.7**	**227.0**	**172.1**	**231.5**	**171.2**	**233.6**	**168.0**	**224.5**	**171.4**	**230.7**	**170.8**	**229.2**
SD	**14.8**	**21.0**	**13.2**	**21.0**	**13.8**	**23.1**	**13.8**	**20.8**	**14.3**	**25.4**	**15.0**	**24.3**
AVR	0.75		0.74		0.73		0.75		0.74		0.75	

All values are in *µ*m. CRAE, central retinal artery equivalent; CRVE, central retinal vein equivalent.

## Data Availability

All data relevant to presented results and conclusions are included in the manuscript.

## References

[1] Broe R., Rasmussen M. L., Frydkjaer-Olsen U. (2014). Retinal vessel calibers predict long-term microvascular complications in type 1 diabetes: the Danish Cohort of Pediatric Diabetes 1987 (DCPD1987). *Diabetes*.

[2] Klein R., Klein B. E., Moss S. E., Wong T. Y., Sharrett A. R. (2006). Retinal vascular caliber in persons with type 2 diabetes: the wisconsin epidemiological study of diabetic retinopathy: XX. *Ophthalmology*.

[3] Blair N. P., Wanek J., Felder A. E. (2017). Retinal oximetry and vessel diameter measurements with a commercially available scanning laser ophthalmoscope in diabetic retinopathy. *Investigative Opthalmology and Visual Science*.

[4] Linsenmeier R. A. (1986). Effects of light and darkness on oxygen distribution and consumption in the cat retina. *Journal of General Physiology*.

[5] Birol G., Wang S., Budzynski E., Wangsa-Wirawan N. D., Linsenmeier R. A. (2007). Oxygen distribution and consumption in the macaque retina. *American Journal of Physiology-Heart and Circulatory Physiology*.

[6] Wang L., Kondo M., Bill A. (1997). Glucose metabolism in cat outer retina. Effects of light and hyperoxia. *Investigative Ophthalmology and Visual Science*.

[7] Wang L., Tornquist P., Bill A. (1997). Glucose metabolism in pig outer retina in light and darkness. *Acta Physiologica Scandinavica*.

[8] Riva C. E, Grunwald J. E., Petrig B. L. (1983). Reactivity of the human retinal circulation to darkness: a laser Doppler velocimetry study. *Investigative Ophthalmology and Visual Science*.

[9] Havelius U., Hansen F., Hindfelt B., Krakau T. (1999). Human ocular vasodynamic changes in light and darkness. *Investigative Ophthalmology and Visual Science*.

[10] Feke G. T., Zuckerman R., Green G. J., Weiter J. J. (1983). Response of human retinal blood flow to light and dark. *Investigative Ophthalmology and Visual Science*.

[11] Jean-Louis S., Lovasik J. V., Kergoat H. (2005). Systemic hyperoxia and retinal vasomotor responses. *Investigative Ophthalmology and Visual Science*.

[12] Dorner G. T., Garhoefer G., Zawinka C., Kiss B., Schmetterer L. (2002). Response of retinal blood flow to CO_2_-breathing in humans. *European Journal of Ophthalmology*.

[13] Polak K., Schmetterer L., Riva C. E. (2002). Influence of flicker frequency on flicker-induced changes of retinal vessel diameter. *Investigative Ophthalmology and Visual Science*.

[14] Arlotte D. V., Perrott R. L., Drasdo N., Owens D. R., North R. V. (2004). The effect of post prandial glucose changes on oscillatory potentials in subjects with type 2 diabetes mellitus. *Documenta Ophthalmologica*.

[15] Holfort S. K., Klemp K., Kofoed P. K., Sander B., Larsen M. (2010). Scotopic electrophysiology of the retina during transient hyperglycemia in type 2 diabetes. *Investigative Ophthalmology and Visual Science*.

[16] Holfort S. K., Jackson G. R., Larsen M. (2010). Dark adaptation during transient hyperglycemia in type 2 diabetes. *Experimental Eye Research*.

[17] Klemp K., Larsen M., Sander B., Vaag A., Brockhoff P. B., Lund-Andersen H. (2004). Effect of short-term hyperglycemia on multifocal electroretinogram in diabetic patients without retinopathy. *Investigative Ophthalmology and Visual Science*.

[18] Fondi K., Wozniak P. A., Howorka K. (2017). Retinal oxygen extraction in individuals with type 1 diabetes with no or mild diabetic retinopathy. *Diabetologia*.

[19] Patel V., Rassam S., Newsom R., Wiek J., Kohner E. (1992). Retinal blood flow in diabetic retinopathy. *BMJ*.

[20] Cuypers M. H., Kasanardjo J. S., Polak B. C. (2000). Retinal blood flow changes in diabetic retinopathy measured with the Heidelberg scanning laser Doppler flowmeter. *Graefe’s Archive for Clinical and Experimental Ophthalmology*.

[21] Yoshida A., Feke G. T., Morales-Stoppello J., Collas G. D., Goger D. G., McMeel J. W. (1983). Retinal blood flow alterations during progression of diabetic retinopathy. *Archives of Ophthalmology*.

[22] Boynton G. E., Stem M. S., Kwark L., Jackson G. R., Farsiu S., Gardner T. W. (2015). Multimodal characterization of proliferative diabetic retinopathy reveals alterations in outer retinal function and structure. *Ophthalmology*.

[23] Taarnhoj N. C., Larsen M., Sander B. (2006). Heritability of retinal vessel diameters and blood pressure: a twin study. *Investigative Opthalmology and Visual Science*.

[24] Jensen B. H., Bram T., Kappelgaard P. (2017). Visual function and retinal vessel diameters during hyperthermia in man. *Acta Ophthalmologica*.

[25] Drobnjak D., Munch I. C., Glumer C. (2016). Retinal vessel diameters and their relationship with cardiovascular risk and all-cause mortality in the inter99 eye study: a 15-year follow-up. *Journal of Ophthalmology*.

[26] Knudtson M. D., Lee K. E., Hubbard L. D., Wong T. Y., Klein R., Klein B. E. (2003). Revised formulas for summarizing retinal vessel diameters. *Current Eye Research*.

[27] Schmetterer L., Muller M., Fasching P. (1997). Renal and ocular hemodynamic effects of insulin. *Diabetes*.

[28] Polak K., Dallinger S., Polska E. (2000). Effects of insulin on retinal and pulsatile choroidal blood flow in humans. *Archives of Ophthalmology*.

[29] Grunwald J. E., Riva C. E., Baine J., Brucker A. J. (1992). Total retinal volumetric blood flow rate in diabetic patients with poor glycemic control. *Investigative Ophthalmology and Visual Science*.

[30] Wang L., Tornquist P., Bill A. (1997). Glucose metabolism of the inner retina in pigs in darkness and light. *Acta Physiologica Scandinavica*.

[31] Arden G. B. (2001). The absence of diabetic retinopathy in patients with retinitis pigmentosa, implications for pathophysiology and possible treatment. *British Journal of Ophthalmology*.

[32] Arden G. B., Gunduz M. K., Kurtenbach A. (2010). A preliminary trial to determine whether prevention of dark adaptation affects the course of early diabetic retinopathy. *Eye*.

[33] Holfort S. K., Norgaard K., Jackson G. R. (2011). Retinal function in relation to improved glycaemic control in type 1 diabetes. *Diabetologia*.

[34] Sander B., Larsen M., Andersen E. W., Lund-Andersen H. (2013). Impact of changes in metabolic control on progression to photocoagulation for clinically significant macular oedema: a 20 year study of type 1 diabetes. *Diabetologia*.

[35] Larsen M., Wang M., Sander B. (2005). Overnight thickness variation in diabetic macular edema. *Investigative Opthalmology and Visual Science*.

[36] Frank R. N., Schulz L., Abe K., Iezzi R. (2004). Temporal variation in diabetic macular edema measured by optical coherence tomography. *Ophthalmology*.

